# Research on Voxel-Based Features Detection and Analysis of Alzheimer’s Disease Using Random Survey Support Vector Machine

**DOI:** 10.3389/fninf.2022.856295

**Published:** 2022-03-28

**Authors:** Xianglian Meng, Yue Wu, Wenjie Liu, Ying Wang, Zhe Xu, Zhuqing Jiao

**Affiliations:** ^1^School of Computer Information and Engineering, Changzhou Institute of Technology, Changzhou, China; ^2^School of Computer Science and Engineering, Changshu Institute of Technology, Changshu, China; ^3^School of Computer Science and Artificial Intelligence, Changzhou University, Changzhou, China

**Keywords:** Alzheimer’s disease, RS-SVM, voxel-based features, gene-level, pathway-level

## Abstract

Alzheimer’s disease (AD) is a degenerative disease of the central nervous system characterized by memory and cognitive dysfunction, as well as abnormal changes in behavior and personality. The research focused on how machine learning classified AD became a recent hotspot. In this study, we proposed a novel voxel-based feature detection framework for AD. Specifically, using 649 voxel-based morphometry (VBM) methods obtained from MRI in Alzheimer’s Disease Neuroimaging Initiative (ADNI), we proposed a feature detection method according to the Random Survey Support Vector Machines (RS-SVM) and combined the research process based on image-, gene-, and pathway-level analysis for AD prediction. Particularly, we constructed 136, 141, and 113 novel voxel-based features for EMCI (early mild cognitive impairment)-HC (healthy control), LMCI (late mild cognitive impairment)-HC, and AD-HC groups, respectively. We applied linear regression model, least absolute shrinkage and selection operator (Lasso), partial least squares (PLS), SVM, and RS-SVM five methods to test and compare the accuracy of these features in these three groups. The prediction accuracy of the AD-HC group using the RS-SVM method was higher than 90%. In addition, we performed functional analysis of the features to explain the biological significance. The experimental results using five machine learning indicate that the identified features are effective for AD and HC classification, the RS-SVM framework has the best classification accuracy, and our strategy can identify important brain regions for AD.

## Introduction

Due to the development of medical technology, the world population has grown steadily, and the elderly population has increased rapidly. It is expected that this trend will continue to accelerate in the next few decades, and the occurrence of senile diseases and the social cost of aging are expected to increase. Alzheimer’s disease (AD) is a brain disease. It is also a progressive disease, meaning that it will get worse over time. It is believed that AD begins 20 years or more before the onset of symptoms (Jiao et al., 2020b). The preclinical stage of AD is crucial for identifying early pathophysiological events and developing interventions for disease improvement. Given that changes in synaptic function occur early in the neurodegenerative process, functional MRI (fMRI) is particularly promising for detecting early changes in brain function (Agosta et al., 2017). MRI has aroused great interest in AD-related research due to its complete noninvasiveness, high availability, high spatial resolution, and good contrast between different soft tissues (Moradi et al., 2015).

Mild cognitive impairment (MCI), known as the early stage of AD, was a disease state of cognitive decline between normal elderly and dementia patients. MCI was divided into early mild cognitive impairment (EMCI) and late mild cognitive impairment (LMCI). Studies had pointed out that if MCI patients were not diagnosed early, the probability of developing AD could be as high as 80% after 6 years, and about two-thirds of AD patients were converted through MCI (Barnes and Yaffe, 2011; Lenhart et al., 2021; Vitali et al., 2021). Using linear mixed models, Vonk et al. (2020) analyzed 2,261 individuals with MCI and non-MCI and found that the neurodegeneration was associated with letter fluency and semantic fluency. Wang et al. (2017) introduced the linear regression classification to classify samples and obtained an accuracy of 97.51% (Zhang et al., 2014). Bi et al. (2020a; 2021b) applied the random forest to identify features associated with AD. Another study showed that the Flash Visual Evoked Potential-P2 latency had AD-specific pathological information (Arruda et al., 2020). Sabuncu et al. (2011) calculated the degree of atrophy of hippocampus and cortical areas and found that the specific cortical thinning and the reduction of hippocampal volume were accelerated in early AD. As the classic analysis methods, the machine learning algorithms brought new research sight to AD-specific biomarkers (Zhang et al., 2015a; Ji et al., 2021; Jiao et al., 2021; Wang et al., 2021). Zhuo et al. (2015) applied a group lasso support vector machine to obtain the AD-specific biomarkers. Patel et al. (2019) developed two XGBoost classification models to classify AD and healthy control (HC). Studies have proved that AD was closely related to brain atrophy and that brain atrophy was mainly reflected in the reduction of cortical surface area, thickness, and gray matter volume and, therefore, gray matter volume, cortical surface area, and average thickness contributed to the pathology of AD patients (Gullett et al., 2021; Lorenzo et al., 2021; Piersson et al., 2021; Talwar et al., 2021).

Despite many efforts, it is still challenging to determine effective AD-specific biomarkers for early diagnosis and prediction of disease progression and requires more research (Bi et al., 2020b; Zhang and Shi, 2020). In our study, we proposed a novel analysis framework based on the Random Survey Support Vector Machines (RS-SVM) for the early detection of AD conversion in MCI patients by using advanced machine learning algorithms and combining voxel-based data with standard neuropsychological test results. First, to obtain the voxel sets, we extracted the differences between AD and HC. Then, we applied the RS-SVM to identify important features that classified EMCI, LMCI, AD, and HC well. Subsequently, we applied several classical methods to construct the analysis frameworks and evaluate the accuracy of these features to classify with EMCI-HC, LMCI-HC, and AD-HC. The experiment results demonstrate that the identified features were effective in classifying AD, the RS-SVM framework performed well, and the identified regions and genes will further our understanding of AD.

## Materials and Methods

**FIGURE 1:**
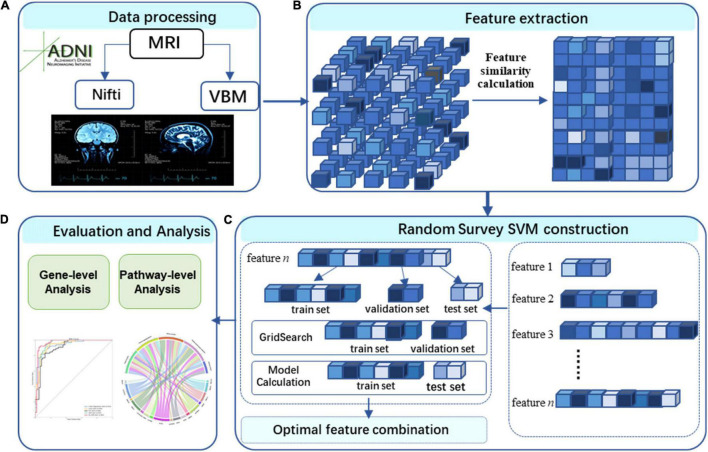
Study workflow. **(A)** We performed image preprocessing on voxel-based measures extracted from structural MRI (VBM-MRI) in the ADNI data set. **(B)** Feature extraction. **(C)** RS- SVM construction. The features were identified by RS-SVM. **(D)** Evaluation and analysis. We assessed the biological significance using gene- and pathway-level analysis.

Figure 1 illustrates the framework of a voxel-based three-level analysis for AD. The framework encompasses data processing (A), features extraction (B), RS-SVM construction (C), and the gene-level analysis using effective chi-square statistic (ECS) method (Li et al., 2019) and pathway-level analysis using the resulting genes (Bu et al., 2021) (D). The novation of this framework is to make the full use of voxel-based data.

### Imaging Data

In this study, we downloaded and analyzed 1,426 participants with genotyping data and MRI scans from the Alzheimer’s Disease Neuroimaging Initiative (ADNI) database (adni.loni.usc.edu). These data include 353 HCs, 273 EMCI, 504 LMCI, and 296 with AD. The characteristics of these participants, including average age and years of education, are shown in Table 1.

**TABLE 1:** Participant characteristics.

Subjects	HC	EMCI	LMCI	AD	p
Number	353	273	504	296	–
Gender (M/F)	187/166	153/120	309/195	166/130	<0.001
Age (mean ± sd)	72.2 ± 7.6	71.3 ± 7.1	74.0 ± 7.6	75.1 ± 5.5	<0.001
Edu (mean ± sd)	16.1 ± 2.7	16.1 ± 2.6	16.0 ± 2.9	16.3 ± 2.6	<0.001

*HC, healthy control; EMCI, early mild cognitive impairment; LMCI, late mild cognitive impairment; AD, Alzheimer’s disease; Edu, education.*

### Random Survey Support Vector Machines-Based Machine Learning Method

#### Data Processing

MRI scans, using voxel-based morphometry (VBM), were aligned and normalized to a T1-weighted template image and the Montreal Neurological Institute (MNI) space, respectively. The gray matter density (GMD) maps were segmented, extracted, and smoothed with an 8-mm FWHM (full width at the half maximum) kernel. The Automatic Anatomical Labeling (AAL) atlas was employed to define the regions of interest (ROIs) and their coordinates (whole brain) (Tzourio-Mazoyer et al., 2002). We then down-sampled the resulting maps to a dimension of 61 × 73 × 61 to reduce the data size for subsequent analysis in EMCI-HC, LMCI-HC, and AD-HC groups.

To extract the differences within the three groups, i.e., A, B, and C, we first performed the weighted process of the two sets of images separately and saved them as matrices *M* and *N* (i.e., *M* for AD group and *N* for HC group). Then, let $\left(v\,m_{i}^{\prime },v\,n_{i}^{\prime }\right)$ represents the vector of two voxels ($v\,m_{i}^{\prime }\in M,v\,n_{i}^{\prime }\in N$), and we obtained a vector $V=\left\{\left(v\,m_{1}^{\prime },v\,n_{1}^{\prime }\right),\left(v\,m_{2}^{\prime },v\,n_{2}^{\prime }\right),\ldots ,\left(v\,m_{k}^{\prime },v\,n_{k}^{\prime }\right)\right\}$ (*k* = 271, 633). Since the voxels ($v\,m_{i}^{\prime }=v\,n_{i}^{\prime }$) in the two groups were meaningless for our research, we deleted these voxels and obtained 64,411 sets of different voxels. We used *V*′ to denote the voxel set.

#### Feature Extraction

The data sets of features were still too many for our final binary classification in EMCI-HC, LMCI-HC, and AD-HC groups. Therefore, we estimated the number of features by calculating the similarity between two rows $\left(v\,m_{i}^{\prime },v\,n_{i}^{\prime }\right)$ and $\left(v\,m_{j}^{\prime },v\,n_{j}^{\prime }\right)$ in *V*′. The similarity of the voxels is given in the following equation:


(1) $$\rho_{i}=\sqrt{{\left(v\,m_{i}^{\prime }-v\,m_{j}^{\prime }\right)}^{2}+{\left(v\,n_{i}^{\prime }-v\,n_{j}^{\prime }\right)}^{2}},\left(v\,m_{i}^{\prime },v\,n_{i}^{\prime }\right),\left(v\,m_{j}^{\prime },v\,n_{j}^{\prime }\right)\in V^{\prime }$$

where $v\,m_{i}^{\prime }$ and $v\,m_{j}^{\prime }$ (*i*,*j* = 1,2,…,64411) are the values of AD group. $v\,n_{i}^{\prime }$ and $v\,n_{j}^{\prime }$ (*i*,*j* = 1,2,…,64411) are the values of HC groups. ρ_*i*_ is the similarity between $\left(v\,m_{i}^{\prime },v\,n_{i}^{\prime }\right)$ and $\left(v\,m_{j}^{\prime },v\,n_{j}^{\prime }\right)$.

For the convenience of calculation, we divided the *V*′ into ten groups and obtained 55 sets of similarity matrices. On this basis, we defined the number of minimal ρ_*i*_ as *C*_*min*_ and the number of maximal ρ_*i*_ as *C*_*max*_. Due to the value of *C*_*min*_ and *C*_*max*_ (*C*_*min*_ = 132, *C*_*max*_ = 21), we defined that the number of features should be in [*C*_*max*_,*C*_*min*_]. Then, we extracted 64,411 features of all subjects from the original MR images to form a 649 × 64,411 matrix as the initial data set.

#### Random Survey Support Vector Machines Construction

To extract the important feature, we proposed a single-kernel SVM model based on random survey. The goal of random survey was the establishment of a random experimental data set. Since the initial data set *X* was a two-dimensional matrix of 649 × 64,411, we selected *l* column from the *X* randomly and constructed a single randomized experimental data set *X*′ (*l* ∈[*C*_*max*_,*C*_*min*_]). At the same time, the set of columns corresponding to each column *l* in each extraction was *R* = {*r*_1_,*r*_2_,…,*r*_*l*_}, which denoted the index of brain loci coordinates. The indices are extracted as follows:


(2) $$\begin{array}{c} u\,\left(k\right)=\left\{u_{1},u_{2},\ldots ,u_{k}\right\},k=64411\\ u^{\prime }\,\left(k\right)=\left\{u_{p},u_{q},\ldots ,u_{r}\right\},r,p,q\in \left[1,64411\right]\\ R=\left\{r_{1}=u_{p},r_{2}=u_{q},\ldots ,r_{l}=u_{g}\right\},g\in \left[1,64411\right] \end{array}$$

After random extraction, we defined the training set:validation set:test set as 6:2:2. The training set was used as an input for training first. The validation set was applied to obtain the optimal hyperparameters and replaced the initial parameters. The remaining 20% was introduced as the test set to calculate the accuracy of the tuned model and to evaluate whether the obtained feature set *R* = {*r*_1_,*r*_2_,…,*r*_*l*_} can be used as the final feature set.

In the classification process of SVM, the input data $X^{\prime }=\left\{X_{1}^{\prime },X_{2}^{\prime },\ldots ,X_{N}^{\prime },\ldots ,X_{M}^{\prime }\right\}$ and the learning objective *y* = {*y*_1_,*y*_2_,…,*y*_*N*_,…,*y*_*M*_} were given, where *N* was the number of EMCI, LMCI, and AD samples, respectively, and *M* was the number of HC. The learning objectives were binary variables *y* = {−1,1}, where -1 represents EMCI, LMCI, and AD, respectively, and 1 represents HC in the three groups. The feature set of the input data was regarded as the hyperplane *D* in decision boundaries to separate the learning targets by positive and negative classes, making the distance ε_*i*_ between any sample and plane ≥1. The hyperplane and the plane distance are defined as follows:


(3) $$\begin{array}{c} D:w^{T}\,X^{\prime }+b=0\\ \varepsilon_{i}=y_{i}\,\left(w^{T}\,X^{\prime }+b\right),\varepsilon_{i}\geq 1 \end{array}$$

where *w* denotes the normal vector of the hyperplane and *b* denotes the intercept of the hyperplane. The decision boundary satisfying this condition actually constructed two parallel hyperplanes *D*_1_,*D*_2_ as interval boundaries to classify the samples (Eq. 4).


(4) $$\begin{array}{c} w^{T}\,X_{i}^{\prime }+b\geq +1\Rightarrow y_{i}=+1\\ w^{T}\,X_{i}^{\prime }+b\leq -1\Rightarrow y_{i}=-1 \end{array}$$

Based on Eq. 4, it could be derived that all samples above the upper interval boundary were positive and those below the lower interval boundary were negative. The distance between the two interval boundaries $d=\frac{2}{||w||}$ was defined as the margin. Since our experimental data *X*′ was selected randomly, there was hyperboloid in the feature set to separate positive and negative classes. Using nonlinear functions, the nonlinear separable problems from the original feature set were mapped to a higher dimensional Hilbert space *H*. The hyperplane, using as the decision boundary, is defined as follows:


(5) $$w^{T}\,\varphi \,\left(X^{\prime }\right)+b=0$$

where φ:*X*′↦H denotes the mapping function. Since the mapping function was complex, it was difficult to calculate the inner product. Therefore, the inner product of the mapping function was defined as kernel functions $k\,\left(X_{1}^{\prime },X_{2}^{\prime }\right)=\varphi \,{\left(X_{1}^{\prime }\right)}^{T}\,\varphi \,\left(X_{2}^{\prime }\right)$ to avoid the explicit operation.

#### Parameter Determination

We used the original linear kernel function of the support vector machine first, and the penalty factor *C* and the kernel parameter gamma were set as default values (*C* = 1 and gamma = 0.5). Then, we applied the training data set and labels to train the model. Subsequently, the hyperparameters were optimized by grid search. The SVM could be transformed into an equivalent quadratic convex optimization problem to solve using the following equation:


(6) $$\begin{array}{c} \min\frac{1}{2}||w|{|}^{2}+C\sum_{i=1}^{M}\varepsilon {}_{i}\\ y_{i}\,\left(w^{T}\,X_{i}^{\prime }+b\right)\geq 1-\varepsilon_{i},\varepsilon_{i}\geq 0 \end{array}$$

### Evaluation Metrics

In this article, the samples were positive and negative, and the results classified had the following cases:

True positive (TP): the positive sample was predicted as a positive sample.

True negative (TN): the negative sample was predicted as a negative sample.

False positive (FP): the negative sample was predicted as a positive sample.

False negative (FN): the positive sample was predicted as a negative sample.

Let *P* denotes the positive sample and *N* denotes the negative sample. We then obtained the following equation:


(7) $$\begin{array}{c} T\,P+F\,N=P\\ F\,P+F\,N=N \end{array}$$

The evaluation metrics used in our research are as follows:

● Accuracy. Accuracy was the number of correctly classified samples divided by the total number of samples (Eq. 8).


(8) $$A\,C\,C=\frac{T\,P+T\,N}{P+N}$$

● Precision. Precision was the proportion of the samples that were actually positive (or negative) divided by samples classified as positive (or negative) (Eq. 9).


(9) $$p\,r\,e\,c\,i\,s\,i\,o\,n=\frac{T\,P}{T\,P+F\,P}$$

● Recall. Recall was the measure of coverage (Eq. 10).


(10) $$r\,e\,c\,a\,l\,l=\frac{T\,P}{T\,P+F\,N}$$

● Comprehensive evaluation indicators (F-Measure). Accuracy and sensitivity sometimes needed to be considered together as given in the following equation:


(11) $$F=\frac{\left(\alpha^{2}+1\right)*P*R}{\alpha^{2}\,\left(P+R\right)}$$

*When*^α = 1^, Eq. 11 is transformed into the following equation as follows:


(12) $$F=\frac{2*P*R}{P+R}$$

### Model Comparison

We used the test set to evaluate the classification ability of 5 machine learning methods, including linear regression model, least absolute shrinkage and selection operator (Lasso) model, partial least squares (PLS) model, SVM model, and RS-SVM model. First, the initial default parameters were applied to each model to train and calculate the evaluation metrics. Then, the grid search algorithm was used to optimize the hyperparameters of the five models. Finally, the hyperparameters were introduced in each model to recalculate the evaluation metrics. The results were used to evaluate the pros and cons of the five models.

Since the RS-SVM model in this article was optimized based on the traditional SVM model, the other three evaluation models were described in detail in this section.

Linear regression model was a statistical analysis method that used regression analysis in mathematical statistics to determine the quantitative relationship between the interdependence of two or more variables.

Given a data set *D* = {(*x*_1_,*y*_1_),(*x*_2_,*y*_2_),…,(*x*_*i*_,*y*_*i*_)}, we learned that a linear model from this data set will reflect the correspondence between *x*_*i*_ and *y*_*i*_ as accurately as possible. The linear regression model, which was a function of linear combination of attributes *x*, could be expressed as follows:


(13) $$f\,\left(x\right)=w_{1}\,x_{1}+w_{2}\,x_{2}+\ldots +w_{i}\,x_{i}+b=W^{T}\,X+b$$

where *W* = {*w*_1_,*w*_2_,…,*w*_*i*_} is column vector, indicating the weight of the corresponding attribute in the prediction result. Eq. 13 was represented as the following equation:


(14) $$f\,\left(x_{i}\right)=w_{i}\,x_{i}+b,f\,\left(x_{i}\right)\approx y_{i}$$

Then it was to find a model such that ∀*i* ∈ [1,*m*] has *f*(*x*_*i*_) as close to *y*_*i*_. Therefore, the sum of the squares of the difference between the predicted value and the real value of each sample is minimized and thus gives the following equation:


(15) $$\left(w^{*},b^{*}\right)=a\,r\,g\,m\,i\,n_{\left(w,b\right)}\,\sum_{i=1}^{m}{\left(f\,\left(x_{i}\right)-y_{i}\right)}^{2}$$

where (w*,b*) is the optimal parameter, and the minimum value of (*w*,*b*) is taken for the above equation.

The Lasso model was a compression estimation method with the idea of reducing the variable set (decreasing order). By constructing a penalty function, it could compress the coefficients of variables and made some regression coefficients become 0, so as to achieve the purpose of variable selection.

Given *n* data samples {(*x*_1_,*y*_1_),(*x*_2_,*y*_2_),…,(*x*_*n*_,*y*_*n*_)} where each *x*_*i*_ ∈ *R^d^* was a *d*-dimensional vector, i.e., each observed data point was composed of the values of *d* variables, and each *y*_*i*_ ∈ *R* was a real value. What we had to do was to find a map *f*:*R^d^*→*R* that minimized the sum of squared errors based on the observed data points. The optimization objective is given as follows:


(16) $$\beta^{*}=\arg m\,i\,n_{\beta }\,\frac{1}{n}\,\sum_{i=0}^{n}{\left(\left(y_{i}-\bar{y}\right)-\beta^{T}\,\left(x_{i}-\bar{x}\right)\right)}^{2}$$

where β ∈ *R^d^* is the optimized coefficient.

If Eq. 16 is expressed in matrix form, denoted by *X* = [*x*_1_;*x*_2_;⋯;*x*_*n*_]^*T*^, where each data point *x*_*i*_ was regarded as a column vector, then *X* ∈ *R*^*n*×*d*^, denoted as *y* = (*y*_1_,*y*_2_,⋯,*y*_*n*_)^*T*^, then the optimization objective in matrix form is given as follows:


(17) $$\beta^{*}=\arg m\,i\,n_{\beta }\,\frac{1}{n}\,{||\text{y-X}\,\beta ||}_{2}^{2}$$

Lasso added the L1 regularization term (see Eq. 18) to make the model avoid over-fitting.


(18) $${||\beta ||}_{1}=\sum_{j=1}^{d}\left|\beta_{j}\right|;1\ll j\ll d$$

Then, the optimization objective function of Lasso is expressed as the following equation:


(19) $$\beta^{*}=\arg m\,i\,n_{\beta }\,\frac{1}{n}\,{||\text{y-X}\,\beta ||}_{2}^{2}+\lambda \,{||\beta ||}_{1}$$

PLS model was a many-to-many linear regression modeling method, i.e., there are multiple independent variables and multiple dependent variables. It found the best functional fit for a set of data by minimizing the sum of squared errors.

The general multivariate underlying model of PLS is given by the following equations:


$$X=T\,P^{T}+E$$

(20) $$Y=U\,Q^{T}+F$$

where *X* is a *n*×*m* prediction matrix, *Y* is a *n*×*p* response matrix; *T* and *U* are *n*×*l* matrices and both of them are the projections of *X* and *Y* in the higher dimensional space; *P* and *Q* are the orthogonal loading matrices of *m*×*l* and *p*×*l*, respectively, and the matrices *E* and *F* are error terms, normally distributed random variables subject to independent and identical distributions. Decompose *X* and *Y* to maximize the covariance between *T* and *U*.

### Gene-Level Analyses

We analyzed the voxel-based features using gene-level analysis. First, quality control (QC) was performed using the PLINK version 1.9 software^1^ (Purcell et al., 2007). We performed genome-wide association studies (GWASs) using the image data and genetic data in whole brain using the linear regression in PLINK. Age, gender, education, and the top 10 principal components from population stratification analysis were included as covariates. A total of 5,574,300 single-nucleotide polymorphisms (SNPs) were obtained by QC. We applied ECS method (Li et al., 2019) to assign SNPs’ to autosomal genes. Then the significant genes was obtained by Bonferroni correction (family-wise error rate *p*-value < 0.05).

### Pathway-Level Analyses

Using the resulting genes, we performed the pathway analysis to assess the biological significance of these features (Bu et al., 2021).KOBAS-I (Bu et al., 2021) pathway analysis tool (KOBAS; bioinfo.org) and the Kyoto Encyclopedia of Genes and Genomes database were applied to pathway analysis of the identified genes (*P* < 0.001).

## Results

In recent studies, machine learning was used to detect the subjects and brain regions of AD (Zhang et al., 2016) and the brain functional statuses of EMCI (Jiao et al., 2020a) and to identify AD and MCI (Zhang et al., 2015b; Wang et al., 2016). In this work, we applied a novel feature extraction method and SVM to obtain the features classified EMCI, LMCI, AD, and HC.

### Comparison of the Five Methods

**FIGURE 2:**
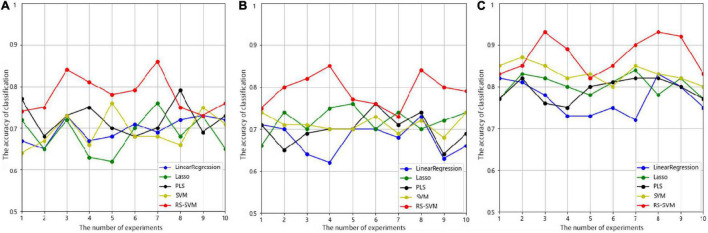
The accuracy curves were obtained through ten experiments for five methods in three groups. **(A)** Prediction accuracy of EMCI-HC group. **(B)** Prediction accuracy of LMCI-HC group. **(C)** Prediction accuracy of AD-HC group.

We employed the test set to evaluate the classification capability of the five methods, and the experiments were repeated 10 times with the selected parameter combination in each method. As shown in Figure 2, the RS-SVM model has the best prediction accuracy. The AD-HC group had more than 90% prediction accuracy, while the other four methods all peaked below 90%. The prediction accuracy of both the EMCI-HC and LMCI-HC groups exceeded 85%, while the peak values of the other four methods were all below 80%. The curves in Figure 2 also showed that RS-SVM had good stability. In ten replicates, the difference in accuracy was less than 10%. These analyses demonstrated the satisfactory classification ability and stability of the RS-SVM model.

Machine learning had been gradually maturing and has been applied to the classification and prediction of AD. We applied the validation set to obtain the optimal parameters and the test set to evaluate the classification capability of the five methods. The evaluation metrics of the five methods implemented in EMCI-HC, LMCI-HC, and AD-HC were shown in Table 2. As shown in Table 2, the RS-SVM has the best accuracy, precision, recall, and F-measure. Only the values of RS-SVM increase with the optimal parameters, and the values of other models are stable. In the AD-HC, EMCI-HC, and LMCI-HC groups, the F-measure of RS-SVM in the validation set were 0.91, 0.86, and 0.85 from high to low. In the AD-HC, EMCI-HC, and LMCI-HC groups, the F-measure of RS-SVM in the test set were 0.93, 0.86, and 0.85 from high to low. This also indicated that the RS-SVM model was scalable, and SVM combined with other schemes have better performance than single SVM. In addition, since the same features were applied to the five models, good results were obtained for all five models (all above 0.8). This proved that the identified features were excellent in the classification of AD and HC and were meaningful for the identification of AD. Therefore, we performed GWAS of these features to analyze their biological significance.

**TABLE 2:** Test results of different models.

Group	Model	Validation set				Test set			
		Accuracy	Precision	Recall	F-Measure	Accuracy	Precision	Recall	F-Measure
EMCI-HC	Linear regression	0.67	0.67	0.67	0.67	0.73	0.73	0.73	0.73
	Lasso	0.79	0.79	0.79	0.79	0.80	0.80	0.80	0.80
	PLS	0.8	0.8	0.8	0.8	0.82	0.81	0.81	0.81
	SVM	0.73	0.73	0.73	0.73	0.76	0.76	0.76	0.76
	**RS-SVM**	**0.86**	**0.86**	**0.86**	**0.86**	**0.86**	**0.86**	**0.86**	**0.86**
LMCI-HC	Linear regression	0.62	0.62	0.62	0.62	0.78	0.78	0.77	0.77
	Lasso	0.80	0.80	0.80	0.80	0.81	0.81	0.81	0.81
	PLS	0.65	0.64	0.65	0.64	0.66	0.65	0.66	0.65
	SVM	0.73	0.73	0.73	0.73	0.74	0.74	0.74	0.74
	**RS-SVM**	**0.85**	**0.85**	**0.85**	**0.85**	**0.85**	**0.85**	**0.85**	**0.85**
AD-HC	Linear regression	0.85	0.85	0.84	0.84	0.84	0.85	0.84	0.84
	Lasso	0.85	0.85	0.85	0.85	0.85	0.85	0.85	0.85
	PLS	0.91	0.92	0.91	0.91	0.91	0.92	0.91	0.91
	SVM	0.87	0.87	0.87	0.87	0.87	0.87	0.87	0.87
	**RS-SVM**	**0.91**	**0.91**	**0.91**	**0.91**	**0.93**	**0.93**	**0.93**	**0.93**

*Bold fonts represented the model and experimental results in this paper.*

### Results of Gene-Level Genome-Wide Association Study

We performed the conditional gene-based association scans on whole genome. All genes with conditional association *P*-values passing Bonferroni correction for family-wise error rate at 0.05 were extracted. We performed the gene-based association analysis by using *P*-values of 113 novel voxel-based features for identifying susceptibility genes of AD. There are 242 genes (corrected *P*-value < 0.001) associated with AD. These top 10 conditionally significant genes are shown in Table 3. Studies have shown that CSMD1 (SNP: rs34464519, CorrectedP: 1.74556E-36) was related to AD (Stepanov et al., 2014; Li et al., 2020; Bi et al., 2021a). RBFOX1 (SNP: rs55642412, CorrectedP: 3.18755E-23) has been found to play a role in neuronal development (Raghavan et al., 2020). PTPRD (SNP: rs62538998, CorrectedP: 1.92988E-19) has been confirmed to be related to AD and MCI in previous studies (Huang et al., 2021). DLGAP2 (SNP: rs72507619, CorrectedP: 3.74049E-17) was found to be predominantly expressed in the brain and associated with a wide variety of neurological disorders (Linthorst et al., 2020). *WWOX* gene has been reported to be a potential mechanism that may be involved in the pathogenesis of AD, focusing on the cell death signaling pathway in neurons (Teng et al., 2012).

**TABLE 3:** Top 10 conditionally significant genes were obtained. Chr represents Chromosome; Gene represents the gene name; CorrectedP represents *P*-value generated by Bonferroni correction.

No.	Chr	Gene	CorrectedP
1	8	CSMD1	1.74556E-36
2	16	RBFOX1	3.18755E-23
3	16	CDH13	1.07119E-20
4	9	PTPRD	1.92988E-19
5	8	DLGAP2	3.74049E-17
6	11	CNTN5	4.81385E-16
7	7	MAGI2	5.93057E-16
8	20	MACROD2	1.50704E-14
9	16	WWOX	1.64798E-14
10	3	CNTN4	1.87567E-13

### Results of Pathway-Level Genome-Wide Association Study

Detecting pathways may provide useful information about the pathogenic molecular mechanism underlying AD. In our work, 70 enriched pathways were identified. The top 10 significant pathways are shown in Table 4. Impaired insulin secretion was associated with higher risk of any dementia and cognitive impairment (Rönnemaa et al., 2008). Oxytocin signaling pathway was neuroprotective to many neurological disorders, such as AD (Almansoub et al., 2020). Vascular smooth muscle contraction was associated with the development of neurodegeneration in AD (Hald et al., 2016).

**TABLE 4:** Top 10 significant pathways.

NO.	Pathways	Corrected *P*-value	Gene
1	Insulin secretion	1.01E-06	PLCB1, PRKCB, PRKCA, CREB5, RYR2, CHRM3, KCNMA1, RAPGEF4, CACNA1C
2	Oxytocin signaling pathway	4.80E-06	PLCB1, PRKAG2, PRKCA, CACNB2, RYR3, RYR2, PRKCB, CACNA1C, ITPR2, CACNA2D3
3	Salivary secretion	7.70E-06	PLCB1, PRKCA, RYR3, PRKCB, CHRM3, KCNMA1, PRKG1, ITPR2
4	Vascular smooth muscle contraction	7.94E-06	PLCB1, CACNA1C, PRKCH, PRKCA, PRKCB, PRKCE, KCNMA1, PRKG1, ITPR2
5	Calcium signaling pathway	1.48E-05	PLCB1, PRKCB, ERBB4, PRKCA, RYR3, RYR2, CHRM3, CACNA1C, ITPR2, PDE1A
6	Glutamatergic synapse	2.08E-05	PLCB1, CACNA1C, PRKCA, GRIK2, PRKCB, DLGAP1, ITPR2, GRM7
7	Morphine addiction	5.00E-05	PRKCA, PDE1A, PRKCB, PDE3A, GABRB3, PDE4D, PDE10A
8	Circadian entrainment	5.56E-05	PLCB1, PRKCB, PRKCA, RYR3, RYR2, CACNA1C, PRKG1
9	Pancreatic secretion	5.56E-05	PLCB1, PRKCB, PRKCA, RYR2, CHRM3, KCNMA1, ITPR2
10	Aldosterone synthesis and secretion	5.56E-05	PLCB1, CACNA1C, PRKCA, CREB5, PRKCB, PRKCE, ITPR2

## Discussion

We proposed a voxel-based three-level analysis framework for AD that extracted the voxel-based ROI, which included the whole brain from MRI. Although the voxel-based research could solve the limitations of the research method based on the ROI, it was more easily affected by the characteristics of high-dimensional data. Feature selection in RS-SVM model solved the dimensional disaster caused by too many attributes. In this work, we identified 136, 141, and 113 MRI features for EMCI-HC, LMCI-HC, and AD-HC groups, respectively.

We performed RS-SVM model to identify important brain regions such as hippocampus, amygdala, angular gyrus, and calcarine sulcus for AD. The hippocampus was located in the midlimbic system of the brain and had an important impact on memory and cognitive function. Many studies had shown that abnormalities in hippocampal volume and function were closely linked to AD. Although many patients had not shown symptoms of AD during MCI period, the temporal lobe located in the inner part of the brain had obvious symptoms. The atrophy of the hippocampus was the most obvious (Adachi et al., 2021; Guo et al., 2021; Zhao et al., 2021). Located at the bottom of the brain, the amygdala was shaped like an almond and was the center of the brain to control and manage emotions. Pathological protein changed in the amygdala affected the occurrence, development, and evolution of AD and led to nerve damage or tissue cell aging (Biechele et al., 2021; Ni, 2021). The angular gyrus was the portion surrounding the end of the superior temporal sulcus in the temporal lobe. Gray matter atrophy gradually spread from the basal ganglia to the angular gyrus, temporal regions, and eventually to the subcortical-cortical network as neurological disease progresses. The angular gyrus was key AD-risk region (Browndyke et al., 2021; Zenke et al., 2021). The calcarine sulcus was located posterior to the medial surface of the hemisphere. Less activation of the bilateral anterior calcarine sulcus was associated with better delayed recall in amnestic MCI patients (Chen et al., 2021; Lejko et al., 2022).

In our work, the EMCI-HC, LMCI-HC, and AD-HC groups were classified in order to increase the specificity and detail of classification and to enable early diagnosis of the disease. In this study, five classification models of linear regression, Lasso, PLS, SVM, and RS-SVM were applied. The prediction accuracy of AD-HC was the highest. EMCI and LMCI represented the middle stage of disease progression. Therefore, the prediction accuracies of EMCI-HC and LMCI-HC were lower than that of AD-HC.

**FIGURE 3:**
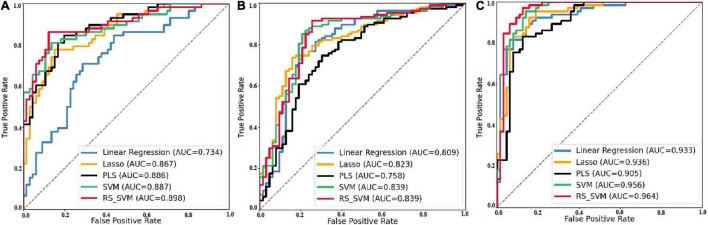
ROC curve of five classification methods for three groups. **(A)** Prediction ROC of EMCI-HC group. **(B)** Prediction ROC of LMCI-HC group. **(C)** Prediction ROC of AD-HC group.

The machine learning was applied to classify AD and HC in previous studies (Zhang and Wang, 2015; Wang et al., 2018; Zhang et al., 2018; Ji et al., 2021; Jiao et al., 2022). For further classification validation, we plot the ROC curves (Figure 3) of five machine learning classification methods for EMCI-HC, LMCI-HC, and AD-HC groups. The AUCs of the RS-SVM for EMCI-HC, LMCI-HC, and AD-HC were 0.898, 0.839, and 0.964, respectively. As can be seen in Table 2, the proposed classification method, based on RS-SVM, consistently outperforms other methods (i.e., linear regression, Lasso, PLS, and SVM). This proved that the framework based on RS-SVM was optimal compared with the other four methods.

With the replication of statistical gene-level GWAS, we obtained *PLCB1*, *PTPRD*, *RYR3*, *CACNA1C*, *KCNMA1*, and *PRKCA* genes. The *PLCB1* gene was implicated in AD pathogenesis (Bastrup et al., 2021). The *PTPRD* gene has been found on the short arm of human chromosome 9. Recent report identified PTPRD association with the extent of neurofibrillary pathology in AD brain specimens (Chibnik et al., 2018; Uhl and Martinez, 2019), and also *RYR3* gene for the RYR which functions to release the stored endoplasmic reticulum calcium ions (Ca^2+^) to increase intracellular Ca^2+^ concentration. The studies demonstrate that altered levels of intracellular Ca^2+^ affect neurodegeneration (Gong et al., 2018; Nilipour et al., 2018). It has been shown that the expression of CACNA1C inhibits the hyperphosphorylation of Tau protein (Jiang et al., 2018).

**FIGURE 4:**
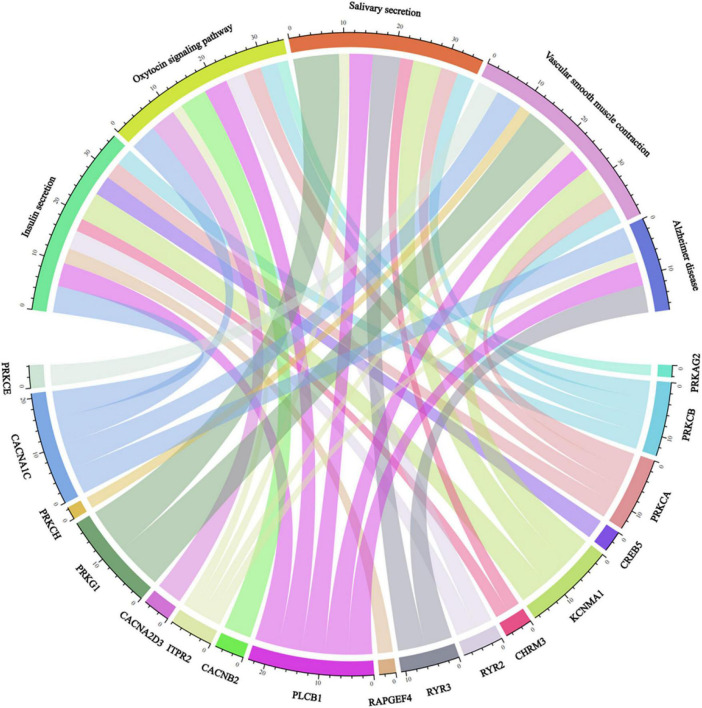
An image showing the relation of genes and pathways.

Some pathways include insulin secretion, oxytocin signaling pathway, salivary secretion, vascular smooth muscle contraction, and AD closely related to genes (Figure 4).

In summary, our proposed framework based on RS-SVM performed well in features constructed, and the framework had good classification performance for EMCI-HC, LMCI-HC, and AD-HC groups. In particular, AD-HC was the best in terms of classification accuracy. Several pathogenic genes and abnormal subregions identified singing this framework are related to AD. Therefore, we speculate that the remaining genes identified could be regarded as the candidate genes for AD. The discoveries in this study provide new candidate genes for AD, and the constructed features can be regarded as a new indicator to distinguish AD from HC.

## Data Availability Statement

Rs-fMRI data were downloaded from the Alzheimer’s Disease Neuroimaging Initiative (ADNI) database (http://adni.loni.usc.edu/). Application for access to the ADNI data can be submitted at http://adni.loni.usc.edu/data-samples/access-data/.

## Ethics Statement

The studies involving human participants were reviewed and approved by Data collection and sharing for this project was funded by the Alzheimer’s Disease Neuroimaging Initiative (ADNI). We applied the acess from ADNI. The patients/participants provided their written informed consent to participate in this study. Written informed consent was obtained from the individual(s) for the publication of any potentially identifiable images or data included in this article.

## Author Contributions

XM, YWu, WL, and ZJ led and supervised research. XM, YWu, and WL designed the research and wrote the article. XM, YWu, and ZX performed features extraction and selection and random survey support vector machines. WL, YWa, and ZX performed data preprocessing and quality control. XM and WL did gene- and pathway-level analysis. All authors reviewed, commented, edited, and approved the manuscript.

## Conflict of Interest

The authors declare that the research was conducted in the absence of any commercial or financial relationships that could be construed as a potential conflict of interest.

## Publisher’s Note

All claims expressed in this article are solely those of the authors and do not necessarily represent those of their affiliated organizations, or those of the publisher, the editors and the reviewers. Any product that may be evaluated in this article, or claim that may be made by its manufacturer, is not guaranteed or endorsed by the publisher.

## Footnotes

1
https://www.cog-genomics.org/plink/1.9/
